# Intraoral Coil Arrays for Single‐Tooth Dental MRI

**DOI:** 10.1002/mrm.70153

**Published:** 2025-10-22

**Authors:** Ali Caglar Özen, Tim Hilgenfeld, Michael Bock

**Affiliations:** ^1^ Division of Medical Physics Department of Diagnostic and Interventional Radiology, University Medical Center Freiburg, Faculty of Medicine, University of Freiburg Freiburg Germany; ^2^ Department of Neuroradiology Heidelberg University Heidelberg Germany

## Abstract

**Purpose:**

MRI offers superior contrast to x‐ray and CBCT that can play an important role in diagnosing dental pathologies. Current extraoral RF coils for MRI in dentistry provide only limited SNR, thus limiting spatial resolution. We propose an intraoral coil (IOC) array that can provide high SNR to achieve a 250 μm isotropic resolution and enable parallel imaging.

**Methods:**

A capacitively decoupled 2‐channel IOC array with a buccal and a lingual element was placed on a bite form covered with silicon putty for subject‐specific fixation. Phantom and in vivo measurements were performed at 3 T to compare the performance of the IOC array to an external single channel loop coil and a single‐channel IOC in terms of sensitivity, image SNR, and g‐factor for 2‐fold acceleration.

**Results:**

IOC array improves SNR by up to 20‐fold compared to the tight‐fit extraoral loop coil (Ø = 4 cm), provides more uniform sensitivity and allows parallel imaging with a g‐factor of less than 1.07 for 2‐fold acceleration. (250 μm)^3^ isotropic resolution of premolar and molar teeth could be realized using a T_1_‐SPACE protocol within 2 min.

**Conclusion:**

Intraoral coil arrays are feasible and offer higher homogeneity and SNR compared to an extraoral loop coil. Moreover, they allow for parallel imaging, which significantly reduces total acquisition time, thereby improving diagnostic potential.

## 1 Introduction

The value of dental MRI has been shown for various applications in endodontics [1, 2, 3], orthodontics [4], craniomaxillofacial surgery [5], and implantology [6]. In dentistry, submillimeter structures need to be imaged such as the root canals and cracks in teeth [7]. To achieve this high resolution in clinically acceptable measurement times, it is advantageous to restrict the imaging FOV to the target volume. FOV restriction can be realized by coils that offer local RF excitation ($B_{1}^{+}$), limited receive sensitivity ($B_{1}^{-}$) tailored to the anatomy, or both. So far, mainly extraoral surface coils have been used [8, 9, 10] which have a distance of about 30–50 mm from the target region (e.g., a molar tooth) [8, 9]. At these large distances a restricted FOV is hardly possible and the receive sensitivity is significantly reduced. Since spatial resolution and signal‐to‐noise ratio (SNR) are inversely proportional, dedicated radiofrequency (RF) coils, intraoral coils (IOCs), are needed, which are smaller in size and can be positioned closer to the target anatomy, thereby increasing the intrinsic SNR [11, 12, 13, 14, 15, 16].

To date, IOCs have been realized with single channel coil designs only. While spatial resolutions as small as 210 μm isotropic were attainable using IOCs [17], the nonetheless necessary acquisition times of 4 min and longer made the measurements, however, susceptible to motion artifacts [18]. This challenge was exacerbated by two factors: First, the natural production of saliva, which triggered the swallowing reflex, and second the physical challenge of having a foreign body inside the mouth. As a result, patient tolerance is significantly lower with IOCs than in conventional MRI exams with external coils. Although the IOCs can be optimized to offer better patient comfort [19] and motion restricting measures can be taken [20], total acquisition times must be reduced to minimize discomfort and the effects of the resulting unwanted motion.

To accelerate dental MRI acquisitions, parallel imaging [21, 22] and compressed sensing [23, 24] combined with iterative [25] or deep‐learning based image reconstruction [26, 27, 28], simultaneous multi‐slice acquisition [29], methods to decrease phase encoding steps, and synthetic imaging methods [30, 31] are directly applicable. However, parallel imaging methods could not be used so far with intraoral coils since no intraoral coil arrays were available. In this study we demonstrate the first 2‐channel IOC array prototype, evaluate its sensitivity and parallel imaging performance.

## 2 Methods

### 2.1 IOC Array Design

**FIGURE 1:**
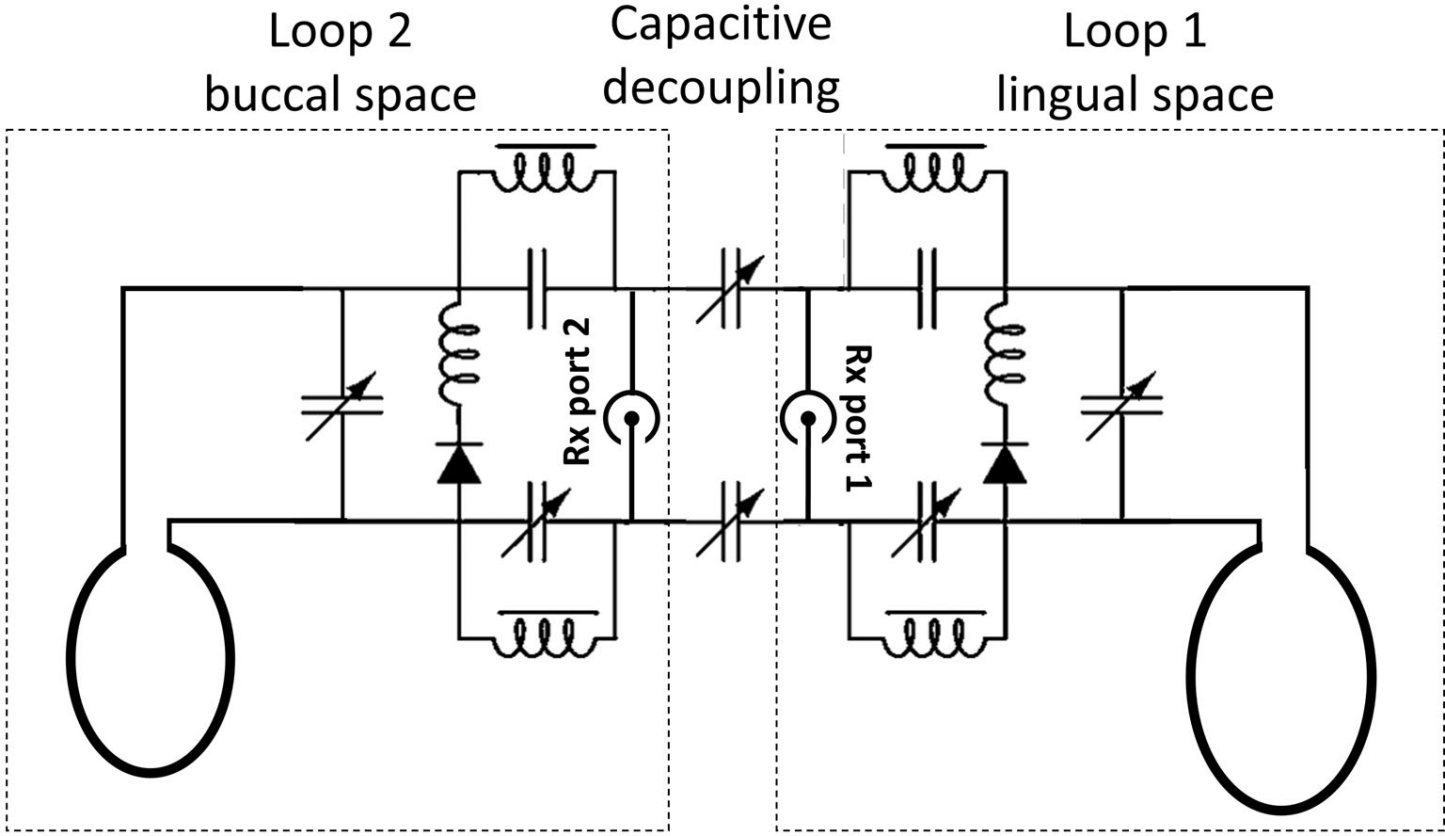
(a) Circuit design of the 2‐channel Rx‐only coil array using a conventional tuning/matching and active detuning interface. Additional decoupling capacitors are used to minimize mutual coupling between the two loops.

**FIGURE 2:**
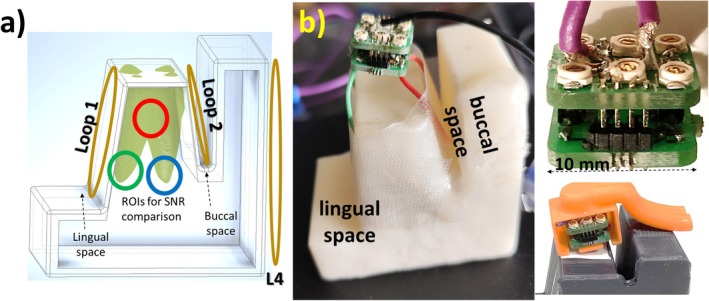
Phantom design for ex vivo characterization for emulating oral anatomy for coil array placement. A sketch of the phantom, photos of the test setup, and insulating cover for in vivo tests are shown. Regions of interests (ROI) are placed at the center of the tooth (pulp), and at the buccal (L1) and palate (L2) dental root canals. (a) A 3D‐printed phantom model and coil placement is shown. As reference, a commercially available loop coil with 4‐cm diameter was used. To fit all the components in a small footprint of 10 × 10 mm^2^, two two‐layer PCBs were stacked on top of each other (b). Coils were soldered on to the bottom layer of the bottom PCB. Coaxial cables from the Rx interface was soldered to the top layer of the upper PCB.

A two‐channel receive‐only IOC array was designed with one buccal element (Loop1) and another lingual element (Loop2, Figure 1). For coil conductors, flexible coaxial cables (Ø = 1.8 mm) were used to form elliptical loops of 75 mm/90 mm circumference for Loop1/Loop2. The elements were decoupled capacitively via two variable capacitors (2–10 pF). Tuning, matching, active detuning and decoupling units were implemented on two stacked double‐layer PCBs of 10 × 10 mm^2^ size (Figure 2b). In total, six variable capacitors (JR100, Knowles Electronics, Itasca, IL) were used for fine adjustment of tuning, matching and decoupling (Figure 2b). Initially, Loop1 and Loop2 were independently tuned to 123.2 MHz and matched to 50 Ω (noise matching for system preamplifiers) with the other element actively detuned. After the variable decoupling capacitors were connected, tuning, matching, and decoupling adjustments were repeated iteratively until the following conditions were met: *S*_11_ and *S*_22_ < −10 dB, and *S*_21_ < −20 dB. To assess loading‐ and position‐dependent variations on *S*_ij_, bench tests were repeated for 9 different positions and three subjects (Figure S1a). IOC array elements were originally adjusted for Subject 1, who participated also in MRI measurements described below.

For electrical insulation a housing was designed to cover the PCBs while leaving the coil conductors out. It was 3D‐printed using dental resin (Figure 2). For testing, a 3D‐printed mandible model phantom was built that emulates, cheek, buccal space, tooth and lingual space. The 3D phantom was filled with water and copper sulfate solution with *ε*_r_ (124 MHz) = 81, *σ*(124 MHz) = 1.93 S/m. With these values, a loading condition was achieved that is similar to the human body, leading to a relative deviation of the S‐parameters of less than 5% (i.e., $\left|\frac{S_{\mathrm{ij},\text{phantom}}}{S_{\mathrm{ij},\text{intraoral}}}-1\right|\leq 0.05$). The dielectric properties were measured with DAKS12 (Zurich Med Tech, Zurich, Switzerland) system. Although the dielectric properties deviate from typical intraoral tissues, for example, $\epsilon_{r,\text{tongue}}=65,\sigma_{\text{tongue}}=0.7$ S/m, $\epsilon_{r,\text{tooth}}=15,\sigma_{\text{toooth}}=0.1$ S/m, $\epsilon_{r,\text{body}\_\text{fluid}}=69,\sigma_{\text{body}\_\text{fluid}}=1.5$ S/m [32, 33, 34], the resulting loading conditions resembled intraoral placement. Source files for the 3D prints of the coil housing, the phantom, and PCB layout files can be downloaded from https://github.com/ozenEEE/UKF_IntraOralCoilArray. 1‐channel coil prototypes of Loop1 and Loop2 were also constructed for SNR comparison with the IOC array. Finally, for in vivo measurements, the IOC array was isolated using a dental impression putty, placed inside a medical hand glove and positioned on the first molar tooth of a 36‐year‐old healthy male volunteer. Volunteer scanning was approved by the Institutional Review Board of the University Medical Center Freiburg (No. 160/2000), and informed written consent was obtained before imaging.

All MRI measurements were conducted at a clinical 3T system (Cima.X, Siemens, Germany). The coils were interfaced using the system's 4‐channel flexible interface. As reference, images were also acquired with the system's 4‐cm diameter loop coil (L4, Siemens) which was placed outside of the mouth. All tested coils were receive‐only coils and the RF excitation was performed using the MRI system's integrated body coil. The detuning network was tested using the body coil as receiver while the IOCs were connected. For SNR comparison, a 2D GRE sequence was repeated 4 times [35]: TR/TE = 10/5 ms, BW = 300 Hz/px, Δ*V* = 0.7 × 0.7 × 0.7 mm^3^, FoV = 140 mm^2^, *α* = 2°. GRAPPA and SENSE was tested for an acceleration factor of 2 with 24 reference lines, and g‐factor maps were calculated. For in vivo measurements the following high‐resolution protocols were applied: fat‐suppressed T1‐SPACE (TR/TE = 700/28 ms, α = 120°, BW = 465 Hz/px, Δ*V* = 0.3 × 0.3 × 0.5 mm^3^, TA = 58 s) and T2‐SPACE (TR/TE/TI = 700/300/210 ms, *α* = 120°, BW = 521 Hz/px, Δ*V* = 0.25 × 0.25 × 0.3 mm^3^, *N*_avg_ = 2, TA = 4:13 m:s), and 3D VIBE (TR/TE = 7.1/2.9 ms, *α* = 8°, BW = 425 Hz/px, Δ*V* = 0.2 × 0.2 × 0.4 mm^3^, TA = 4:09 min:s).

To test potential RF‐induced heating of the IOC array, the steps introduced in [36] were followed: Hotspot detection and temperature measurements. Temperature measurements were performed during an RF‐pulse‐only pulse sequence with system‐reported whole‐body SAR value of 4 W/kg. An HEC‐gel phantom with *ε*_r_ (124 MHz) = 79.2, *σ*(124 MHz) = 0.61 S/m was prepared [37]. The IOC array was immersed in the phantom with the coil plane orthogonal to ***B***_0_ and temperature rise near the coil was monitored at different locations using four fiber‐optic temperature probes (FOTEMP6‐19, Optocon AG, Dresden, Germany) as shown in Figure S2. At least one FOTP was positioned at the hotspot determined using E field mapping as described in [36, 38, 39, 40, 41]. Since the E field sensor is mounted on a 2D translational stage, E field mapping was performed for three different orientations of the IOC array (Figure S3).

## 3 Results

The tuning and matching measurements of Loop1 and Loop2 yielded *S*_11/22_ = 14.9/17.5 dB and *S*_21_ = −27.2 ± 3.3 dB, where the variations in *S*_21_ originates from the changes in loading conditions between phantom and in vivo. The Q ratios were *Q*_ratio_(Loop1) = 79/55 = 1,44 and *Q*_ratio_(Loop2) = 85/67 = 1,27. Although Q_ratio_ was lower than 2 for both coils, indicating that the coil losses are comparable to sample noise, with the decreasing circumference, SNR was still 1.6‐fold higher in 75 mm coil than the 90 mm coil.

**FIGURE 3:**
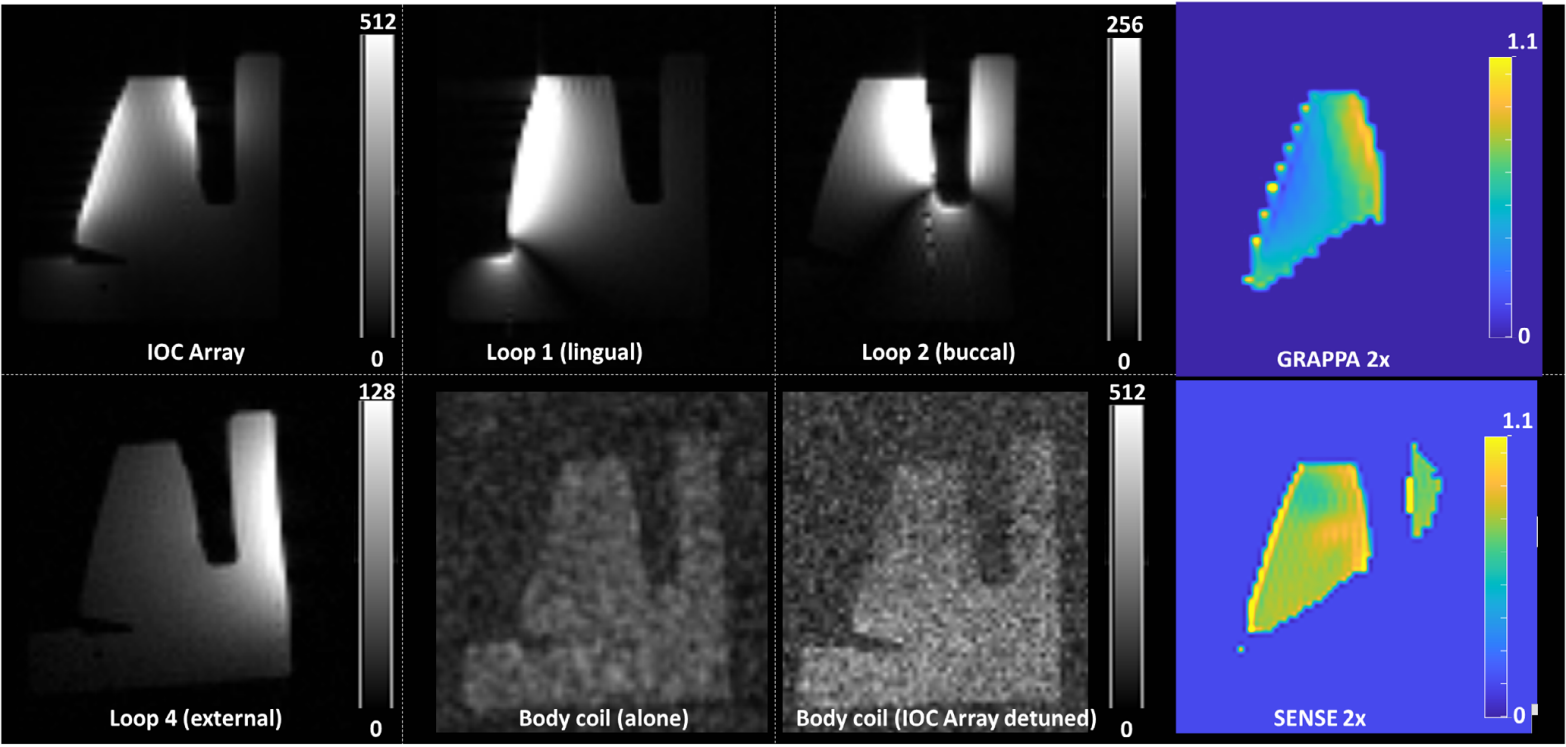
SNR maps of IOC array, individual loop coil elements of the array (Loop1 and Loop2), external loop coil (Loop 4) and body coil images without the IOC array and when the IOC array was positioned but detuned. g‐factor maps for 2‐fold acceleration using SENSE and GRAPPA techniques.

Figure 3 shows coronal GRE images of the phantom for all coils. Compared to Loop1 and Loop2 alone, the IOC array compensates for the limited sensitivity profile at the center of the tooth. Single coil measurements show that the buccal Loop2 element provides an up to 4‐fold higher SNR than the lingual Loop1 element at the center of the tooth close to the crown (Table 1). Around the apices of the roots, however, Loop1 outperforms Loop2, due to the increased sensitivity profile from the larger coil diameter. Compared to the external loop coil L4, the IOC array an achieved an SNR gain between 5.3‐ and 20.2‐fold (Table 1). The SNR penalty during 2‐fold accelerated parallel imaging was less with the GRAPPA than SENSE, especially at the center of the phantom, and both methods yielded g‐factors of less than 1.07. In the body coil image negligible coupling with the IOC array is seen, corresponding to a maximum of 11% SNR difference than the image without the IOC array, indicating a very effective detuning.

**TABLE 1:** SNR comparison of the selected coils at the selected region of interests (ROIs) defined in the phantom images.

Coil/ROI	SNR_ROI_ = mean(SNR_Voxel_)		
	Red (crown)	Green (root‐lingual)	Blue (root‐buccal)
Loop 1 (lingual)	84.4	160.0	339.4
Loop 2 (buccal)	410.2	98.8	64.0
IOC array	529.5	285.6	568.8
Loop 4 (external)	40.8	54.2	30.9
Body coil alone	6.7	7.3	6.9
Body coil (IOC array detuned)	6.9	7.4	7.2

E field maps yielded relatively low peak E field values of up to 24 V/m (Figure S3). Hotspots were positioned at the feed ports, distributed asymmetrically. During the temperature measurements at the hot spots, no significant heating was observed, with a maximum temperature rise of 0.1°C ± 0.1°C measured using the high‐SAR protocol.

Position‐dependent variations in *S*_ij_ were below 5 dB with *S*_11_ = −14.0 ± 2.2, *S*_22_ = −12.9 ± 1.9 and *S*_21_ = −21.5 ± 2.6 dB for Subject 1 (Figure S1). *S*_11_, *S*_22_ and *S*_21_ had standard deviations of up to 4.0, 2.7 and 4.2 dB, respectively. When the IOC array was placed at the incisors, where the distance between the coil elements was minimum, *S*_11_, *S*_22_ and *S*_21_ among all subjects reached a maximum value of −8.6, −9.2 and −12.7 dB, respectively.

**FIGURE 4:**
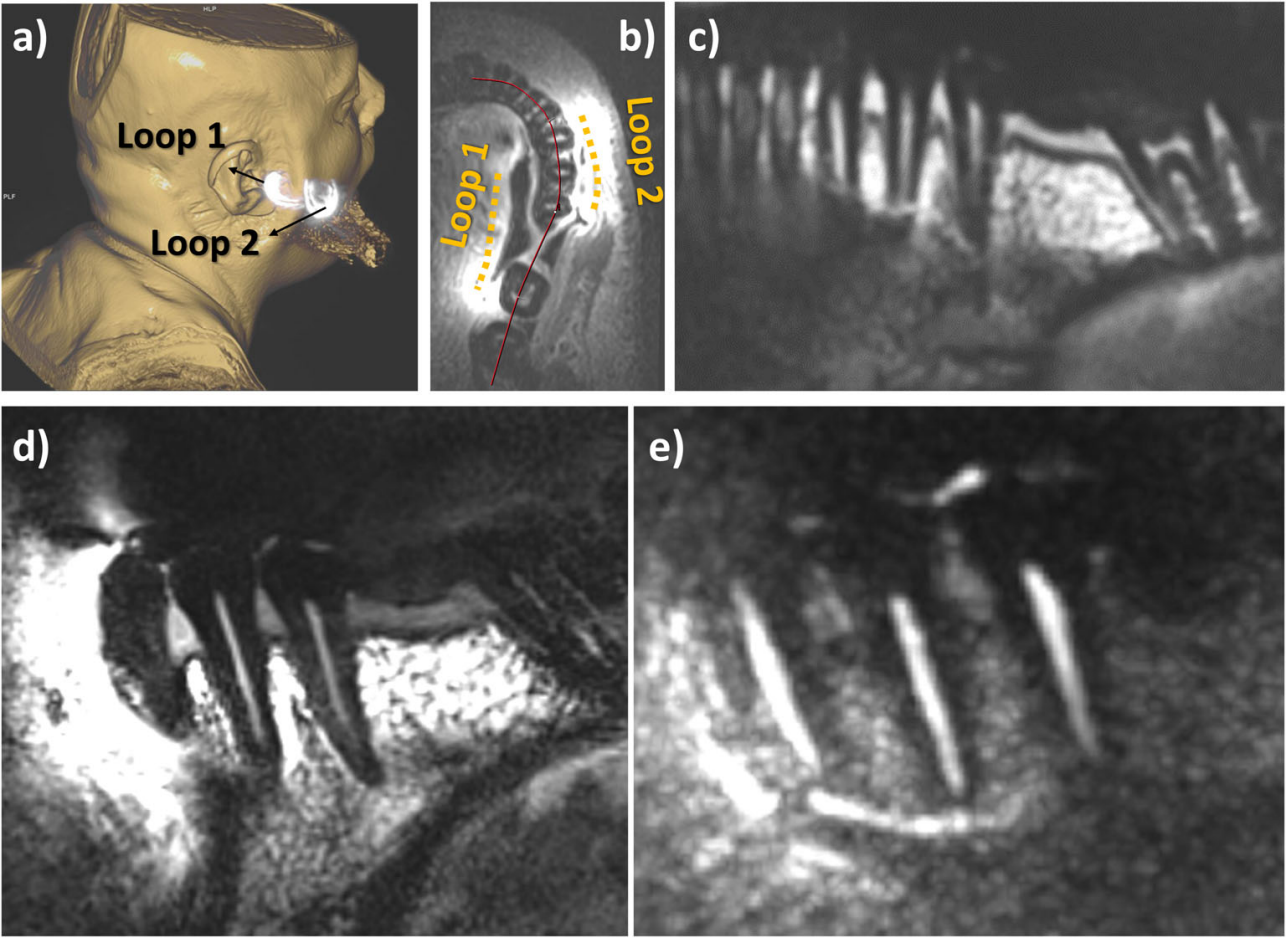
In vivo images of premolar and molar teeth at the left mandible of a healthy volunteer (M‐36). A surface rendering from maximum intensity projection image obtained using the VIBE sequence for localization of the coil (a). An axial slice from the 1‐mm‐resolution VIBE sequence showing the relative position of the coil conductors (a) and a panoramic view of the canals (c) along the red line drawn in (b). T1‐ (d) and T2‐weighted SPACE images with 300 μm‐in‐plane‐resolution (e).

In Figure 4, an in vivo images of premolar and molar teeth of a healthy volunteer is shown. In the maximum intensity projection image (Figure 4a), motion artifacts in left–right direction are visible. SNR values above 100 was obtained for voxel sizes of 0.25 × 0.25 × 0.3 mm^3^ even at the apices of the roots with a maximum deviation of 17% from the peak value along the canal. Coil positions were slightly off set compared to the phantom positioning unintentionally, yet a large overlap is still visible (Figure 4a,b), resulting in a full coverage of four tooth regions in the lower left jaw (tooth region 34–37; Figure 4c–e). Both T_1_‐ (Figure 4d) and T_2_‐weighted SPACE (Figure 4e) images successfully resolved the canal structure, while T_1_‐weighted image provided also the supporting alveolar bone structures.

## 4 Discussion

In this study, we introduced an intraoral coil array for dental MRI and for the first time demonstrated in vivo. An SNR gain of 19‐fold is obtained compared to a tight‐fit extraoral flexible coil. Low SNR penalty at the acceleration factor of 2 can be used either to enhance the resolution or to reduce the total acquisition time.

Previous intraoral coils suffered from reduced sensitivity towards the apices of the roots [12, 13, 14, 15, 16]. For single‐tooth coils, buccal space is limited and the coil is always positioned above the roots. Here, the IOC array covers the roots mainly by the lingual element (Figure 3). Although the buccal element shows higher SNR in the selected ROIs due to the smaller coil size, the lingual element contributes significantly to the overall SNR performance of the IOC array (Figure 3). As the coils were not perfectly aligned around one tooth, additional methods for lingual positioning of the IOC elements will need to be integrated (e.g., modified dental bites), which might also prove useful for inductively coupled coils [15].

Currently, the coil array uses 6 variable capacitors (Figures 1 and 2), which is the main constraint for its size, and which makes this prototype tedious to wear. Use of smaller fixed capacitors might address this issue and should be investigated to evaluate their tolerance to variations in loading and mechanical stress. Note that, after sealing the IOC array, it was not retuned between the imaging sessions, indicating a certain degree of robustness against minor variations in loading conditions. Extending this coil concept to a higher number of coil elements in the array can considerably increase the SNR which might allow for even higher acceleration factors in parallel acquisition techniques. Shortening the acquisition times will reduce motion artifacts and will increase patient compliance thus ultimately leading to improved image quality in endodontics. The concept can be potentially translated to lower field strengths, currently envisioned for dental MRI applications, to compensate for the SNR loss due to lower B_0_ [42].

*Q*_ratio_ values suggests that IOC array elements operate in sample‐noise dominated regime, which is not preferred in conventional RF coil design for clinical MRI. Lower *Q*_ratio_, however, does not directly imply a lower intrinsic SNR and the optimal loop coil radius should be selected according to the target depth of sensitivity [43]. The SNR gain offered by the smaller coil element encourages even smaller coil elements forming three or four channel IOC arrays for single tooth MRI. Although feasibility of three and four channel IOC arrays needs to be further investigated. Currently, the elements Loop1 and Loop2 are designed to be placed on opposite sides of the tooth leading to a maximal geometric coupling. Here, this is reduced by capacitive decoupling, but with more coil elements on both sides of the tooth also the geometric coupling could be reduced by arranging the lingual and buccal elements not directly collinearly, but by shifting them relative to each other. This would reduce noise coupling and would enable parallel imaging acceleration in more than one direction.

The in vivo measurements were performed using a custom‐made head rest that is simply formed by two PVC support plates on the sides that fixes the head with the help of support foams. Although this setup restricts the head motion from left to right direction to some extent, motion artifacts were visible in some of the sequences (Figure 4a). MRI with intraoral coils at high resolutions require additional measures for motion management of not only the head, but also the jaw, especially the mandible and even the tongue. Although no readily available solutions exist to overcome this challenge, motion tracking to omit/re‐acquire k space lines that are affected by the motion could be applied to dental MRI. Bite‐form fixation has also been used in a few studies with intraoral coils [12, 14, 16]. An MR‐compatible salive management system could also reduce swallowing during measurement with intraoral coils. Patient specific bite forms and coil formers could also enhance comfort and thus reduce unwanted motion.

Although current MR safety standards and guidelines do not explicitly mention IOCs, the guidelines for active implanted devices can be used to assess the safety of IOCs [44]. Here, we used a hot spot detection followed by temperature measurements as in [36, 40]. In general, Tier 3 and 4 of ISO 10974 guidelines could also be applied to assess MR safety of IOCs. However, in Tier 4, electromagnetic simulations are performed, which is time‐consuming for IOCs as small components like capacitors increase the computational burden. Unless the capacitors are modeled as lumped elements, the Tier 4 is less feasible. If capacitors are modeled as lumped elements, their influence on the accuracy of the absolute SAR values is unknown. Alternatively, a transfer function approach could be applied using the experimentally detected hot spot as the focal point of the scattered electric fields [38, 40, 41]. A comparison between the different safety assessments will be studied in the future. Faster imaging protocol thanks to the acceleration achieved by IOC arrays allows acquiring more data before hitting the SAR limits by shortening total acquisition time.

The IOC array was manually coated with a liquid UV‐sensitive resin (Dental Model Resin V3, Formlabs GmbH, Berlin, Germany) and resin powder (Lucitone 199, Dentsply Sirona, Dentsply Detrey GmbH, Konstanz, Germany) mixture. To form more uniform insulation layers, the viscosity of the insulation material can be lowered such that the coil can be dipped into a resin bath; however, this imposes a further constraint on the choice of the coating material and mixture. Combination of a core insulation layer with partial patient specific coating could result in more comfortable and stable coil placement. For example, if a registration of the patient's bite as a mold to apply the insulation layer, the teeth could be engaged perfectly to the coil surface, thus eliminating the need for biting on the coil to hold it and ensuring the relationship between the teeth and coil element are appropriately positioned. The current MR properties of the insulation were satisfactory; however, an insulation material with a lower dielectric loss could further improve coil performance.

Smaller diameter coils offer higher sensitivity (Figure S4), yet, the smallest diameter coil that is available as a clinical equipment was the 4‐cm‐diameter loop coil in this study. An optimization of extraoral coils could enhance their performance, so that the SNR gain in this study might decrease when compared to such an optimized extraoral coil. To benchmark the performance of the IOC array in vivo, we used only clinically available coils (Figure S5), as custom coils require additional ethics approval procedures and optimization of extraoral coils is beyond the scope of this study. Similarly, testing different IOC prototypes across several volunteers is needed to reach a statistical conclusion on the advantages of the IOC arrays.

This study was performed at 3T since it is a moderately accessible clinical field strength. It should also be applicable at lower field strengths, for example, at the clinically most available *B*_0_ = 1.5 T. Optimal field strength for dental applications is however yet to be determined, as lower fields provide the advantages of longer $T_{2}^{*}$, shorter T1, and reduced metal artifacts, as well as potentially higher accessibility [45, 46, 47, 48].

## 5 Conclusion

Intraoral coils are attractive alternatives to external coils for dental MRI. The proposed IOC array offered an over 20‐fold higher SNR than the extraoral coil, leading to substantial advantages in terms of acquisition time and/or spatial resolution. Moreover, it allowed for the first time to use advanced acceleration methods in dental MRI such as deep learning‐based image reconstruction and image synthesis, which could accelerate image acquisition beyond conventional parallel imaging and minimize patient discomfort and the resulting motion artifacts in high resolution dental MRI.

## Supporting information


**FIGURE S1:** Position‐ and subject‐dependent variability of $S_{\mathrm{ij}}$ parameters of the IOC array. For the single subject measurements, reflection coefficients $S_{11}$ and $S_{22}$ remained below −10 dB for all positions with the lowest being at positions 3 and 4. When positioned at the incisors (number 5) the coupling was higher due to the reduced distance between the coil elements; therefore, reflection coefficients were also higher. Subject‐dependent variation was below 4.0/2.7/4.2 dB for $S_{11}$/$S_{22}$/$S_{21}$. Position dependent variations were below 2.5/2.6/3.2 dB for $S_{11}$/$S_{22}$/$S_{21}$.
**Figure S2:** Temperature probe positioning and placement inside gel phantom. IOC array did not cause any significant heating in detuned state. Maximum heating was 0.7°C ± 0.1°C for Loop2 coil positioned on the side in position P2, as plotted on the right hand side.
**Figure S3:** Electric field mapping was performed using a custom‐made electrooptic sensor placed in saline. 2D E field maps of the coil elements were measured in both detuned and tuned states first when the coil elements are facing each other and when the coil elements are positioned on a planar surface. A maximum E field of 55 V/m was measured when the coils were tuned. No significant E field was coupled when the coils were detuned.
**Figure S4:** To maximize signal‐to‐noise‐ratio (SNR) sample‐to‐coil distance must be minimized. SNR of simulations of intraoral coils were compared to extraoral coils that fit tightly on cheek and is positioned 5 mm away, i.e., External 1 and 2, respectively. Intraoral coils positioned on the buccal and lingual side of the teeth provide up to 13‐fold higher SNR, which is crucial for high resolution dental applications.
**Figure S5:** In vivo images of premolar and molar teeth at the left mandible of a healthy volunteer (M‐36) acquired using the extraoral L4 coil, which was placed approximately at the same position as the IOC array. A surface rendering from maximum intensity projection image obtained using the VIBE sequence for localization of the coil (a). An axial slice from the 1‐mm‐resolution VIBE image (b). T1‐ (c) and T2‐weighted SPACE images with 300 μm‐in‐plane‐resolution (d).

## Data Availability

The data that support the findings of this study are available from the corresponding author upon reasonable request.
