# Supplementary material for: Intraoral Coil Arrays for Single‐Tooth Dental MRI
Source: Magn Reson Med. 2025 Oct 22;95(3):1858–65. doi: 10.1002/mrm.70153 (PMC12746383; doi:10.1002/mrm.70153)
Supplement: Supplementary file 1 — FIGURE S1: Position‐ and subject‐dependent variability of Sij parameters of the IOC array. For the single subject measurements, reflection coefficients S11 and S22 remained below −10 dB for all positions with the lowest being at positions 3 and 4. When positioned at the incisors (number 5) the coupling was higher due to the reduced distance between the coil elements; therefore, reflection coefficients were also higher. Subject‐dependent variation was below 4.0/2.7/4.2 dB for S11/S22/S21. Position dependent variations were below 2.5/2.6/3.2 dB for S11/S22/S21. Figure S2: Temperature probe positioning and placement inside gel phantom. IOC array did not cause any significant heating in detuned state. Maximum heating was 0.7°C ± 0.1°C for Loop2 coil positioned on the side in position P2, as plotted on the right hand side. Figure S3: Electric field mapping was performed using a custom‐made electrooptic sensor placed in saline. 2D E field maps of the coil elements were measured in both detuned and tuned states first when the coil elements are facing each other and when the coil elements are positioned on a planar surface. A maximum E field of 55 V/m was measured when the coils were tuned. No significant E field was coupled when the coils were detuned. Figure S4: To maximize signal‐to‐noise‐ratio (SNR) sample‐to‐coil distance must be minimized. SNR of simulations of intraoral coils were compared to extraoral coils that fit tightly on cheek and is positioned 5 mm away, i.e., External 1 and 2, respectively. Intraoral coils positioned on the buccal and lingual side of the teeth provide up to 13‐fold higher SNR, which is crucial for high resolution dental applications. Figure S5: In vivo images of premolar and molar teeth at the left mandible of a healthy volunteer (M‐36) acquired using the extraoral L4 coil, which was placed approximately at the same position as the IOC array. A surface rendering from maximum intensity projection image obtained using the VIBE sequence for loc [file MRM-95-1858-s001.pdf]

Intraoral Coil Arrays for Single-Tooth Dental MRI

Ali Caglar Özen[1], Tim Hilgenfeld[2], Michael Bock[1]

[1]Division of Medical Physics, Dept. Radiology, Univ. Medical Center Freiburg, Univ. Freiburg, Freiburg, Germany
[2]Dept. Neuroradiology, Heidelberg University, Heidelberg, Germany

Supplementary Material

Corresponding author: Ali Caglar Özen
Killianstr.5a · 79106 Freiburg
Telefon: +49 761 270-93910
Telefax: +49 761 270-93900
ali.oezen@uniklinik-freiburg.de

Position- and Subject-dependence of S Parameters

IOC array was originally fine-tuned and matched at the first molar position for Subject 1. To assess variations in S_{ij}, 9 different positions were measured. The same coil was also tested on two additional volunteers. The results are summarized in Supporting Information Figure S1.

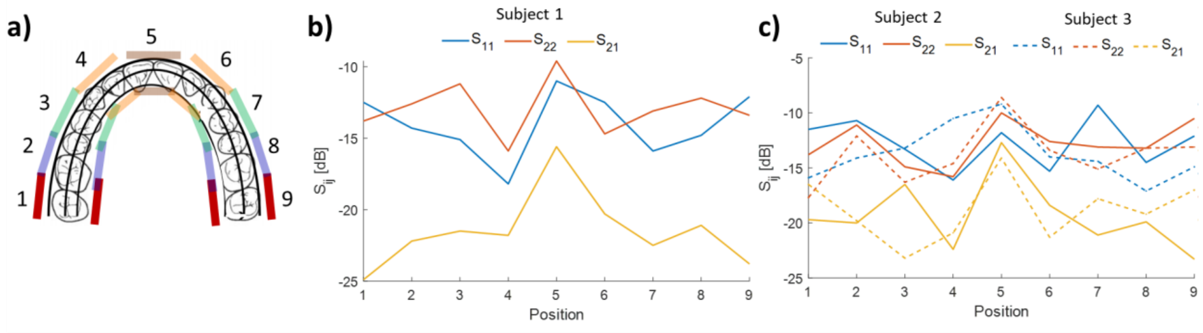

Supporting Information Figure S1: *Position- and subject-dependent variability of S_{ij} parameters of the IOC array. For the single subject measurements, reflection coefficients S_{11} and S_{22} remained below -10 dB for all positions with the lowest being at positions 3 and 4. When positioned at the incisors (number 5) the coupling was higher due to the reduced distance between the coil elements; therefore, reflection coefficients were also higher. Subject-dependent variation was below 4.0/2.7/4.2 dB for S_{11}/S_{22}/S_{21}. Position dependent variations were below 2.5/2.6/3.2 dB for S_{11}/S_{22}/S_{21}.*

RF-Safety Evaluation

Two-step RF safety evaluation was performed with hot spot mapping using high resolution E field mapping and temperature measurements at the hot spots during high SAR protocols both in tuned, i.e., resonant and detuned states.

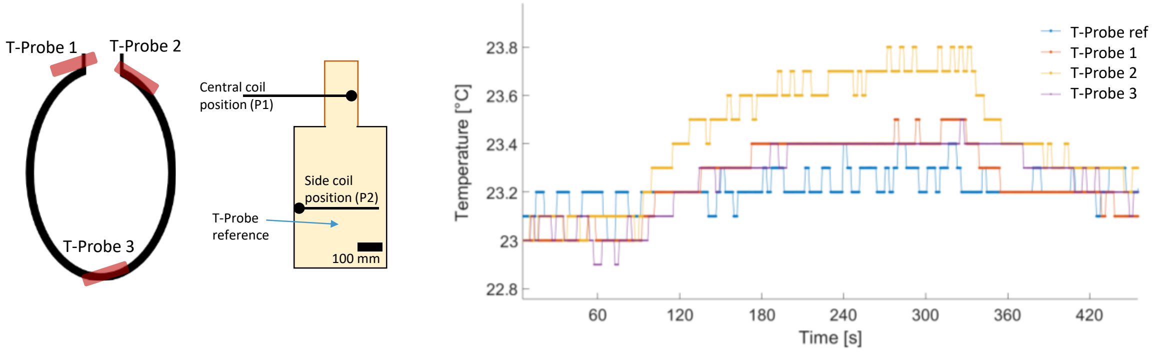

Supporting Information Figure S2: *Temperature probe positioning and placement inside gel phantom. IOC array did not cause any significant heating in detuned state. Maximum heating was 0.7 ± 0.1 °C for Loop2 coil positioned on the side in position P2, as plotted on the right hand side.*

Electric field mapping was performed using an electrooptic sensor mounted on a 2D translational stage at 1 mm spatial resolution. Individual loops were mapped by placing IOC array in a way that one of the loops is positioned on top. Another measurement was performed by placing both of the loops on a flat surface to map the E field around both coil conductors simultaneously. During E field measurements, a dipole antenna tuned to 123.2 MHz was used as the excitor. Coils and the excitors were immersed in saline with ε_r = 81, σ = 0.46 S/m. Detuning of the coils was achieved actively using an external power supply.

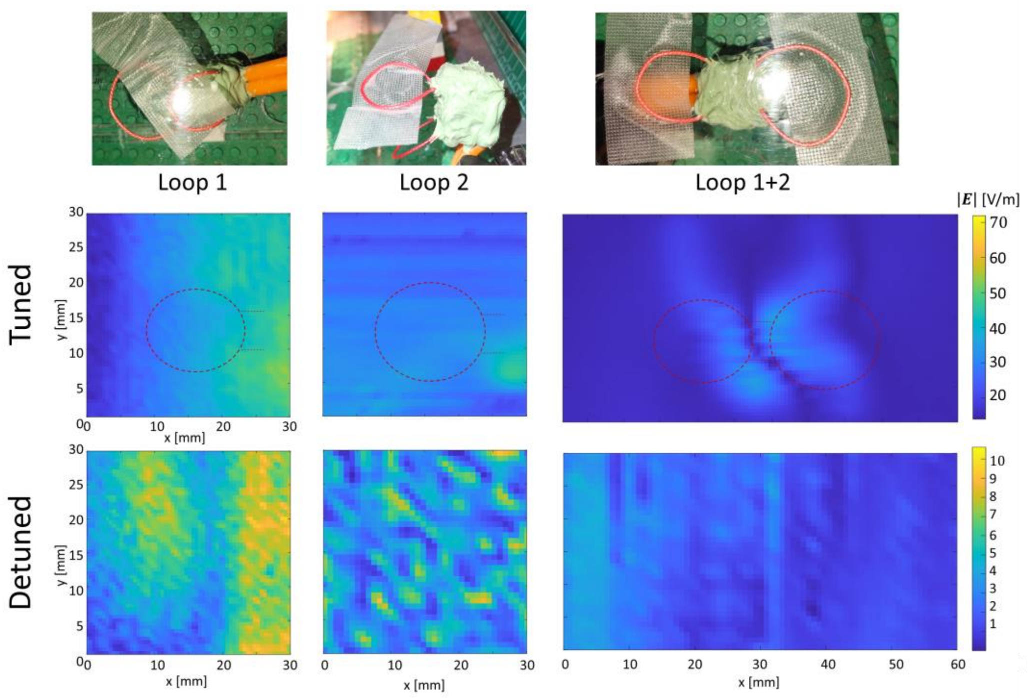

Supporting Information Figure S3: *Electric field mapping was performed using a custom-made electrooptic sensor placed in saline. 2D E field maps of the coil elements were measured in both detuned and tuned states first when the coil elements are facing each other and when the coil elements are positioned on a planar surface. A maximum E field of 55 V/m was measured when the coils were tuned. No significant E field was coupled when the coils were detuned.*

Intraoral vs. Extraoral Coil Simulations

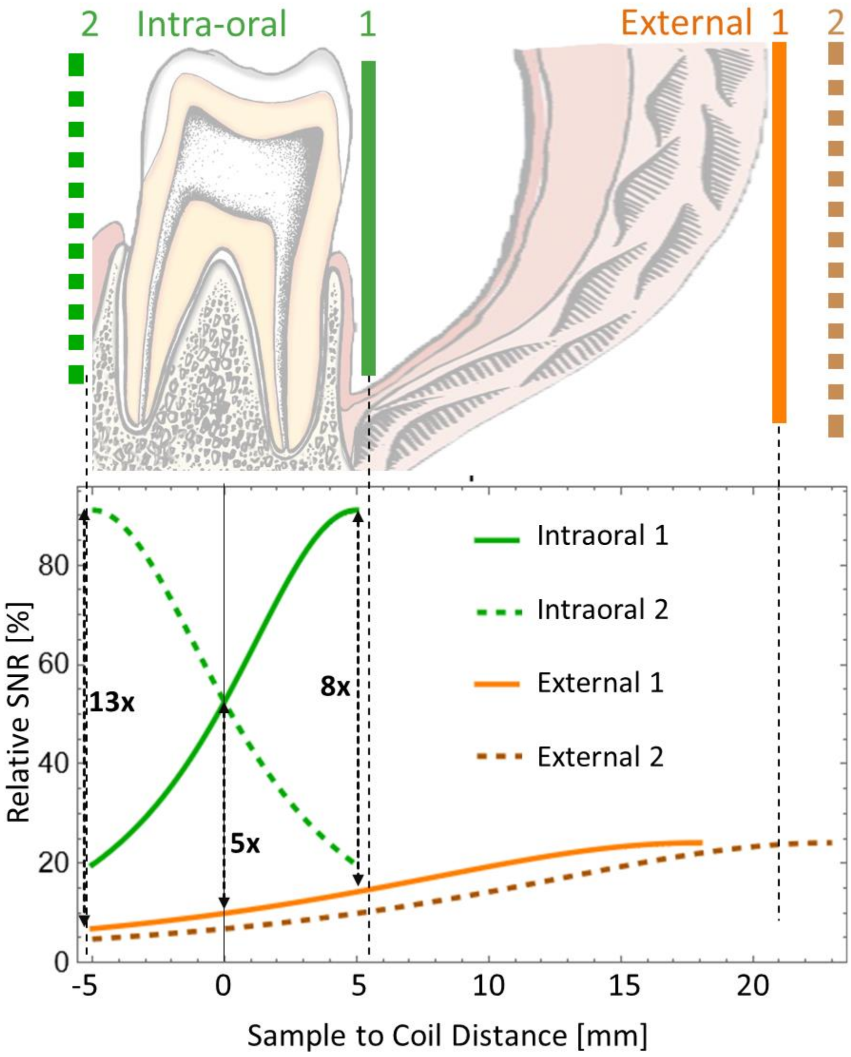

Supporting Information Figure S4: *To maximize signal-to-noise-ratio (SNR) sample-to-coil distance must be minimized. SNR of simulations of intraoral coils were compared to extraoral coils that fit tightly on cheek and is positioned 5 mm away, i.e., External 1 and 2, respectively. Intraoral coils positioned on the buccal and lingual side of the teeth provide up to 13-fold higher SNR, which is crucial for high resolution dental applications.*

In vivo Dental MRI Using the Commercially Available Extraoral Loop 4 Coil (L4)

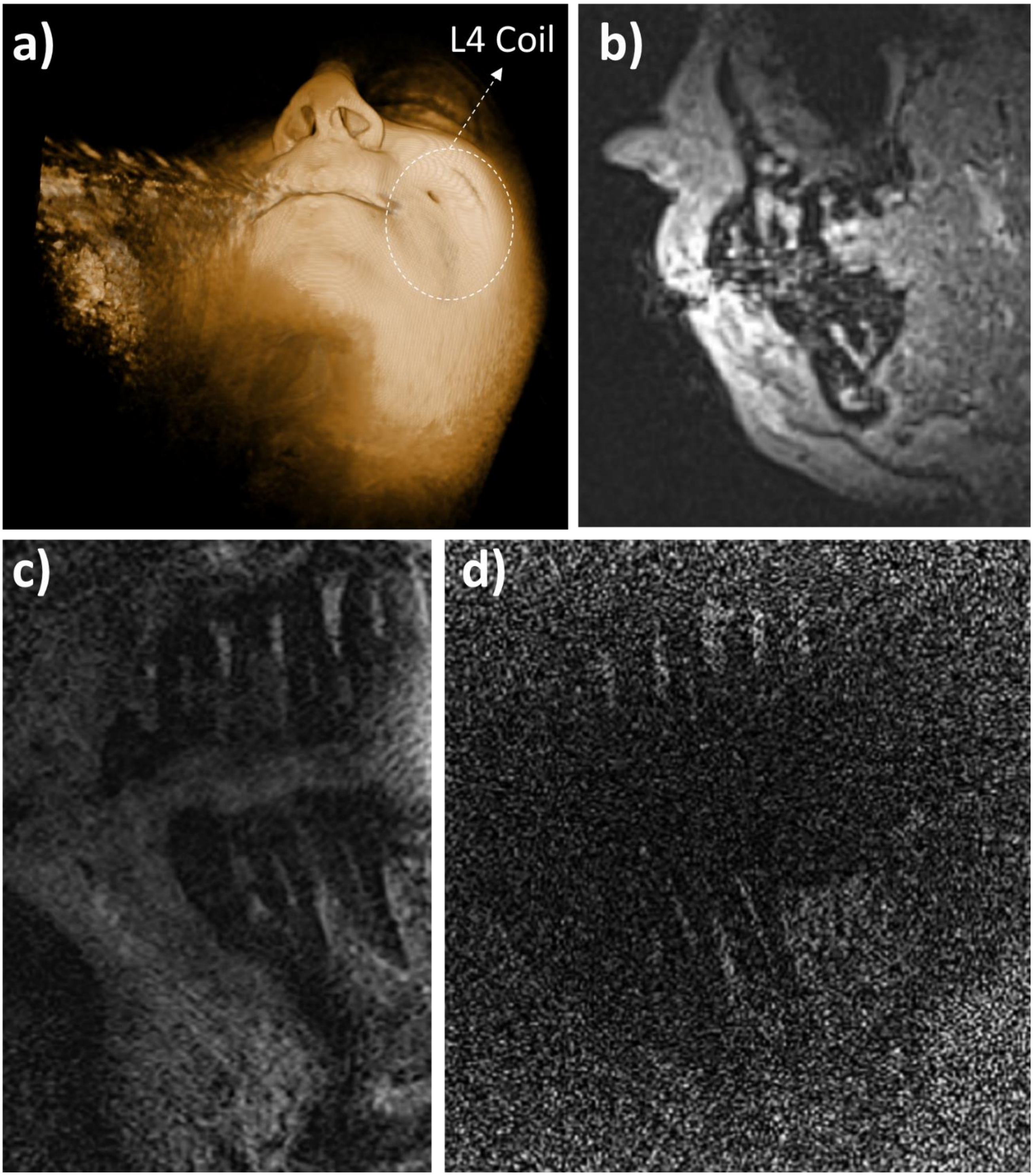

Supporting Information Figure S5: *In vivo images of premolar and molar teeth at the left mandible of a healthy volunteer (M-36) acquired using the extraoral L4 coil, which was placed approximately at the same position as the IOC array. A surface rendering from maximum intensity projection image obtained using the VIBE sequence for localization of the coil (a). An axial slice from the 1-mm-resolution VIBE image (b). T1- (c) and T2-weighted SPACE images with 300 μm-in-plane-resolution (d).*
